# LncRNA expression signature identified using genome-wide transcriptomic profiling to predict lymph node metastasis in patients with stage T1 and T2 gastric cancer

**DOI:** 10.1007/s10120-023-01428-8

**Published:** 2023-09-10

**Authors:** Zhe-bin Dong, Han-ting Xiang, Heng-miao Wu, Xian-lei Cai, Zheng-wei Chen, Sang-sang Chen, Yi-Chen He, Hong Li, Wei-ming Yu, Chao Liang

**Affiliations:** Department of General Surgery, The Affiliated Lihuili Hospital, Ningbo University, 57 Xingning Road, Ningbo, 315000 People’s Republic of China

**Keywords:** Bioinformatics, Biomarkers, Gastric cancer, Lymph node metastasis, LncRNA signature

## Abstract

**Background:**

Lymph node (LN) status is vital to evaluate the curative potential of relatively early gastric cancer (GC; T1–T2) treatment (endoscopic or surgery). Currently, there is a lack of robust and convenient methods to identify LN metastasis before therapeutic decision-making.

**Methods:**

Genome-wide expression profiles of long noncoding RNA (lncRNA) in primary T1 gastric cancer data from The Cancer Genome Atlas (TCGA) was used to identify lncRNA expression signature capable of detecting LN metastasis of GC and establish a 10-lncRNA risk-prediction model based on deep learning. The performance of the lncRNA panel in diagnosing LN metastasis was evaluated both in silico and clinical validation methods. In silico validation was conducted using TCGA and Asian Cancer Research Group (ACRG) datasets. Clinical validation was performed on T1 and T2 patients, and the panel’s efficacy was compared with that of traditional tumor markers and computed tomography (CT) scans.

**Results:**

Profiling of genome-wide RNA expression identified a panel of lncRNA to predict LN metastasis in T1 stage gastric cancer (AUC = 0.961). A 10-lncRNA risk-prediction model was then constructed, which was validated successfully in T1 and T2 datasets (TCGA, AUC = 0.852; ACRG, AUC = 0.834). Thereafter, the clinical performance of the lncRNA panel was validated in clinical cohorts (T1, AUC = 0.812; T2, AUC = 0.805; T1 + T2, AUC = 0.764). Notably, the panel demonstrated significantly better performance compared with CT and traditional tumor markers.

**Conclusions:**

The novel 10-lncRNA could diagnose LN metastasis robustly in relatively early gastric cancer (T1–T2), with promising clinical potential.

**Supplementary Information:**

The online version contains supplementary material available at 10.1007/s10120-023-01428-8.

## Background

Globally, gastric cancer (GC) is fifth in the list of most common cancers and ranks fourth as the most common cause of cancer death [1]. Lymph node (LN) metastasis is a major clinical feature of GC, which influences the poor prognosis of patients with GC [2]. Even for early GC, the 10-year survival rates of patients with or without LN metastasis are significantly different, at 72 and 92%, respectively [3]. Accurate evaluation of LN status in patients with GC before treatment is critical to evaluate the degree of disease and improve treatment strategies. Currently, the diagnosis of LN metastasis is carried out mainly using conventional tumor markers (carcinoembryonic antigen (CEA) and carbohydrate antigen 19–9 (CA19-9)) and computed imaging methods (computed tomography (CT) and positron emission tomography with CT (PET-CT)). Unfortunately, these methods show poor performance to clinically identify LN and frequently demonstrate poor correlation and high error rates [4, 5]. Thus, there is an urgent need for more accurate and reliable detection methods to identify LN metastasis in GC, which might be used to enhance the prognosis of patients with GC significantly.

Gastric cancer is still treated using surgery and endoscopic resection. Currently, Asian and European guidelines identify endoscopic submucosal dissection (ESD) and endoscopic mucosal resection (EMR) as the first choice treatments for most cases of early GC (cT1a) and are considered to be safe and definitive treatments [6, 7]. However, patients who are considered to be at risk of LN metastasis after endoscopic surgery will undergo additional radical surgery, because of submucosal invasion (T1b), large tumor size, and poor differentiation [8]. Unfortunately, pathological examination of these post-gastrectomy tissues, especially from early GC, revealed that only about 20% of patients were identified as having LN metastasis [9, 10]. In past decades, the optimal extent of lymphadenectomy has also been discussed extensively in the field of surgery. With the development of precision medicine, for patients with GC with cT1-T2N0M0 status, laparoscopic sentinel node navigation surgery (LSNNS) was proposed for stomach preservation, which showed no difference in 3-year overall survival (3y-OS) and 3-year disease free survival (3y-DFS) compared with laparoscopic standard D2 gastrectomy, but resulted in better long-term quality of life and nutritional status [11, 12]. Prospective evaluation of sentinel lymph node navigation surgery for relatively early GC (T1–T2) is a current development trend of function-preserving, personalized, and minimized gastrectomy [12–14]. However, LSNNS is based on a comprehensive assessment of the LN status of patients, which is a challenge for its practical application. The lack of accurate and reliable detection of preoperative LN metastasis status means that many patients have experienced unnecessary overtreatment, which also limits the beneficial development of precision medicine.

It is highlighted by genome-wide association studies in cancer that single-nucleotide polymorphisms (SNPs) are related to cancer risk and more than 80% of cancer-associated SNPs occur in noncoding regions of the genome [15]. In addition, most somatic mutations, copy number alterations, and cancer-related SNPs are related to ncRNAs. Long noncoding RNAs (lncRNAs) account for the majority of human ncRNAs (approximately 76%) and maintain homogenous expression within and between tumor tissues [16, 17]. Functionally, long noncoding RNAs are found in sense or antisense orientation to protein-coding genes, in introns of protein-coding genes or in intergenic regions of the genome, and mediate positive or negative regulation [18]. Presently, the number of disease-related lncrnas identified by experiments is less than 1% of the identified sites, and its biological function needs to be further explored.

Herein, transcriptome-wide expression profiles of long noncoding RNA (lncRNA) were analyzed comprehensively and systematically, and a 10-lncRNA panel was established to identify GC LN metastasis (T1 and T2). We verified the effectiveness of the panel in independent databases and clinical tissue samples. The performance of the lncRNA panel was also compared with that of CEA, CA19-9, and CT, highlighting the value of this panel in predicting LN metastasis of T1 and T2 GC. The lncRNA panel could function as the basis for clinical decision-making for patients with GC.

## Methods

### Public datasets and the identification of the gene-expression signature

To identify an lncRNA expression signature for the detection of lymph node (LN) metastasis in gastric cancer (GC), the study used genome-wide expression profiles of lncRNAs from primary tumors with and without LN metastasis, which were obtained from The Cancer Genome Atlas (TCGA) database. Only pathological T1 and T2 RNA-sequencing (RNA-Seq) data were used for further analysis. The T1 data were from 15 LN metastasis negative (LNN) and 5 LN metastasis positive (LNP) samples, and the T2 data were from 34 LNN and 48 LNP samples. The processed TCGA level 3 RNA-Seq data for GC were obtained from the Firehose Broad GDAC portal [19]. Independent validation data were downloaded from the Asian Cancer Research Group (ACRG). In the gene-level RNA-Seq by Expectation–Maximization (RSEM files), we converted the scaled estimates to transcripts per million (TPM) by multiplying them by 10^6^, and then carrying out log2-transformation. We filtered all lncRNA expression levels from the TCGA and ACRG processed data according to the human gene annotation file [20] (https://ftp.ensembl.org/pub/release97/gtf/homo_sapiens/Homo_sapiens.GRCh38.97.chr.gtf.gz). Then, logistic regression analysis was performed using the Logistic Regression (LR) function from Pytorch [21] (citation https://arxiv.org/abs/1912.01703). Feature importance was estimated using coefficients from the LR model. To assess the lncRNA panel's diagnostic accuracy, the selected lncRNA features were used to construct a multivariate LR model, followed by calculation of the area under the receiver operator characteristic (ROC) curve (AUC) values. Ultimately, the probability of each patient being identified as LNP was used as the basis to calculate the risk scores. The flowchart of this study is shown in Supplementary Fig. 1.

### Clinical cohort evaluation

To validate the identified lncRNA markers and for clinical training, we enrolled three independent patient cohorts comprising 245 cases in total. Cohort 1 consisted of 20 surgically resected GC specimens from 8 LNP patients and 12 LNN patients. Cohort 2 included 98 patients (LNP = 19, LNN = 79). Cohort 3 included 127 patients (LNP = 38, LNN = 89). Patients in the clinical cohorts were treated at the Lihuili Hospital affiliated to Ningbo University (China). These patients had biopsy-proven primary GC and underwent curative surgery between December 2017 and January 2022. During surgery, we obtained tissues samples from a representative malignant lesion located in the surgically excised stomach specimen. The tissue samples were added with RNAstore (CWBIO, Shanghai, China), frozen rapidly in liquid nitrogen, and stored at − 80 ºC. The summarized characteristics of the patients in the clinical cohorts are shown in Table 1 and Fig. 2A.

**Table 1:** Clinical characteristics of the patients in cohorts 2 and 3

Characteristics	Clinical cohort 2 (*n* = 98)		Clinical cohort 3 (*n* = 127)	
	LN positive (*n* = 19)	LN negative (*n* = 79)	LN positive (*n* = 38)	LN negative (*n* = 89)
Age (years)	69.53 ± 6.141	68.62 ± 7.550	69.42 ± 8.465	69.00 ± 0.769
Sex				
Male	11	40	23	49
Female	8	39	15	40
CEA (ng/ml)	4.226 ± 3.046	2.890 ± 1.597	4.750 ± 3.307	3.794 ± 2.098
Positive	7	8	10	21
Negative	12	71	28	68
CA19-9 (U/ml)				
Positive	8	8	9	14
Negative	11	71	29	75
CT				
Positive	5	9	12	10
Negative	14	70	26	79
T stage				
T1	19	79	0	0
T2	0	0	38	89
N stage				
N0	0	79	0	89
N1	16	0	25	0
N2	3	0	10	0
N3	0	0	3	0
LV invasion				
Positive	15	8	30	35
Negative	4	71	8	54
Venous invasion				
Positive	12	15	26	34
Negative	7	64	12	55

The CEA cutoff value is 5 ng/ml; the CA19-9 cutoff value is 37 U/ml

*LN* lymph node, *CEA* carcinoembryonic antigen, *CA19-9* carbohydrate antigen 19–9, *CT* computed tomography, *T stage* tumor stage, *N stage* node stage, *LV* lymphovascular

### RNA extraction and quantitative real-time reverse transcription PCR (qRT-PCR) analysis

An RNeasy mini kit (QIAGEN, Hilden, Germany) was used to isolate total RNA from frozen surgical tissues, following the supplier's guidelines. The RT-PCR step of the qRT-PCR protocol was carried out using a SensiFAST probe Lo-ROX Kit (Bioline, London, UK) and the qPCR step used the QuantStudio 6 Flex Real-Time PCR System (Applied Biosystems, Foster City, CA, USA). Assay reproducibility was ensured via multiple techniques, such as including appropriate controls, excluding specimens with poor RNA quality, and the analysis of multiple replicates carried out at various time points. The QuantStudio 6 Flex Real-Time PCR System Software (Applied Biosystems) was used to assess gene expression. The expression level of *ACTB* (encoding beta actin) was used to determine and correct the relative expression of target genes, employing the 2^–Δ*C*t^ method. In this method, Δ*C*t is the difference in cycle threshold (*C*_t_) values between *ACTB* and the gene of interest. The data were then log_2_ transformed. Supplementary Table 1 details the PCR primers used.

### Statistical evaluations

The method of *DeLong* [22] was used to assess the statistically significant differences among the ROC curves. Python (version 3.8, https://www.python.org/) was used to carry out the statistical analyses. Two-tailed *t* test-determined *p* values less than 0.05 indicated statistical significance.

### LncRNA enrichment analysis

For lncRNA enrichment analysis, we used the website application constructed by Chen et al. [23] (https://doi.org/10.1093/nar/gkaa806). The data were visualized on a histogram and bubble chart using ggplot2 [24].

## Results

### Genome-wide lncRNA expression profiles identified a 10-lncRNA panel to predict LN metastasis in T1 and T2 stage GC

**Fig. 1:**
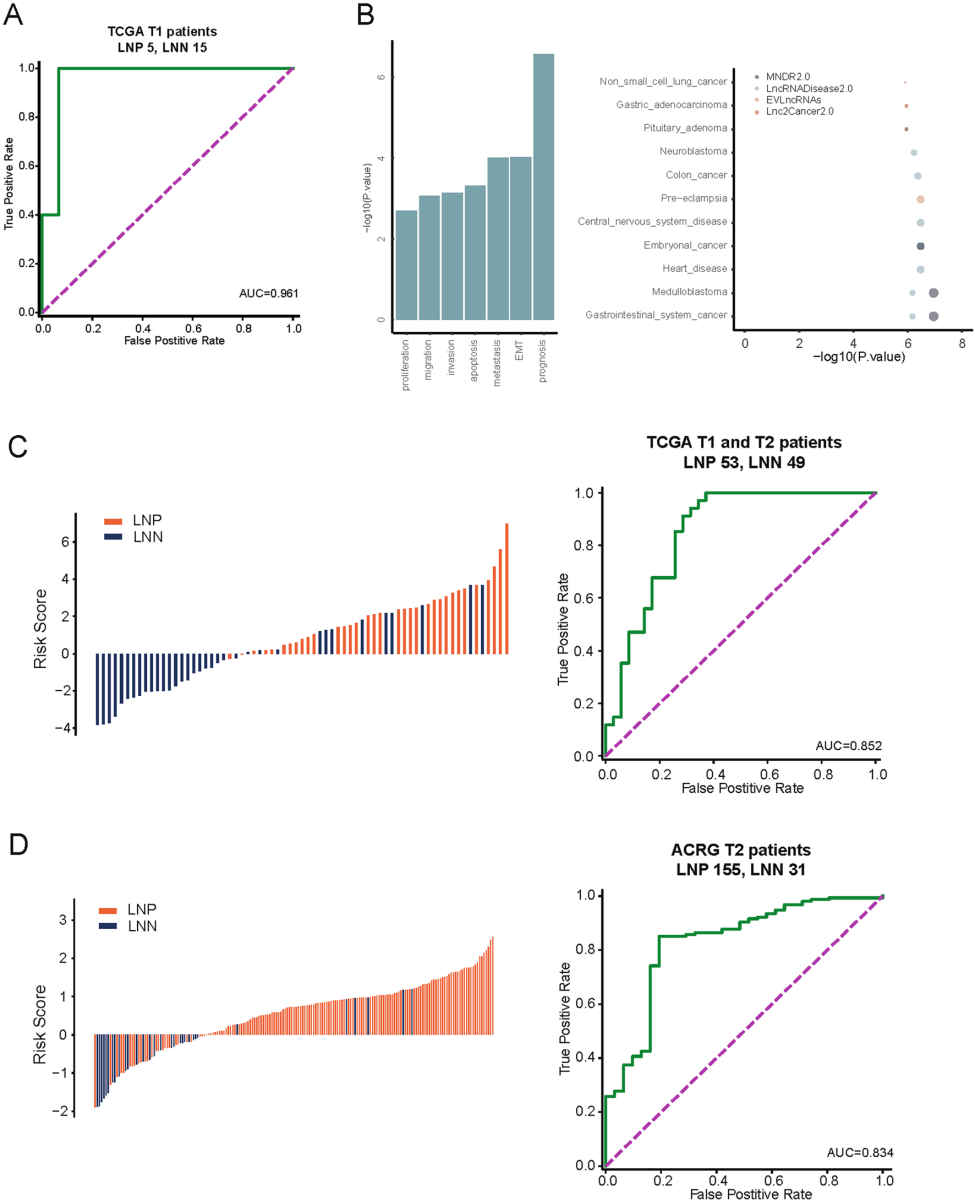
The long noncoding RNA (lncRNA) expression-based signature to identify lymph node metastasis in T1 and T2 stage gastric cancer

First, we systematically and comprehensively analyzed RNA-seq expression profiling data from patients with GC at T1 stage in the TCGA database, which included 5 LNP patients and 15 LNN patients, to identify an lncRNA expression signature to diagnose patients with T1 stage GC with LN metastasis using deep learning model. The validation of the holdout dataset demonstrated that the model could distinguish patients with LNP GC from those with LNN GC (AUC = 0.961, Fig. 1A). To make the lncRNA signature more practical and suitable for clinical use, we prioritized lncRNAs resulted in a 10-lncRNA signature for further validation based on the feature importance in logistic regression, which included five relatively highly expressed lncRNAs (*H19*, *CECR7*, *HOTAIR*, *FAM66D*, *C22orf34*) and five lncRNAs with relatively low expression (*TTTY15*, *TTTY14*, *TP53TG1*, *HAR1A*, *C10orf95*) in the LNP versus LNN comparison. In addition, tumor functional enrichment analysis was carried out for this panel. The results revealed that this 10-lncRNA panel was closely related to tumor prognosis, epithelial–mesenchyme transition (EMT), and metastasis, and was specific for gastrointestinal system cancer (Fig. 1B).

Considering that both T1N0M0 and T2N0M0 are stage I gastric cancer, and the significance of T2 lesions in the current precision medicine of GC, the predictive accuracy of the 10-lncRNA panel was also validated in the dataset containing patients with T2 GC. Notably, individual lncRNAs showed limited performance in external independent datasets, while the integration of all 10 lncRNAs demonstrated significant performance. Multivariate LR analysis was then used to obtain a 10-lncRNA risk-prediction model: risk score =  − 0.141 * *TTTY15* − 0.140 * *TTTY14* − 0.117 * *TP53TG1* − 0.100 * *HAR1A* − 0.074 * *C10orf95* + 0.166 * *H19* + 0.212 * *CECR7* + 0.222 * *HOTAIR* + 0.226 * *FAM66D* + 0.236 * *C220rf34*. Using a larger TCGA cohort (including 20 T1 and 82 T2 patients) and a cohort of 186 T2 patients from the ACRG (Fig. 1C, D), the risk model could differentiate LNP from LNN patients (AUC = 0.85, Fig. 1C; AUC = 0.83, Fig. 1D). This 10-lncRNA panel exhibited a robust performance in two independent validated datasets, highlighting its potential for diagnostic prediction of LN metastasis in patients with T1 and T2 stage GC.

A. Receiver operating characteristic (ROC) curves revealing the diagnostic performance of the model to distinguish lymph node-positive (LNP) and lymph node-negative (LNN) patients in The Cancer Genome Atlas (TCGA) T1 samples. B. Histogram and bubble diagrams showing the enrichment analysis of cancer hallmark and disease in the 10-lncRNA panel. C. The lymph node (LN) risk scores divided by LN status in the T1 and T2 cohorts from the TCGA, shown as a waterfall diagram, and a receiver operating characteristic (ROC) curve showing how the 10-lncRNA risk-prediction model performed in diagnosing patients with T1 and T2 stage disease in the TCGA data. D. The LN risk scores divided by LN status in the T2 cohort from Asian Cancer Research Group (ACRG), shown as a waterfall diagram, and a ROC curve showing how the 10-lncRNA risk-prediction model performed in diagnosing patients T2 stage disease in the ACRG data.

### Validation of the 10-lncRNA risk-prediction model to identify lymph node metastasis in independent clinical cohorts

**Fig. 2:**
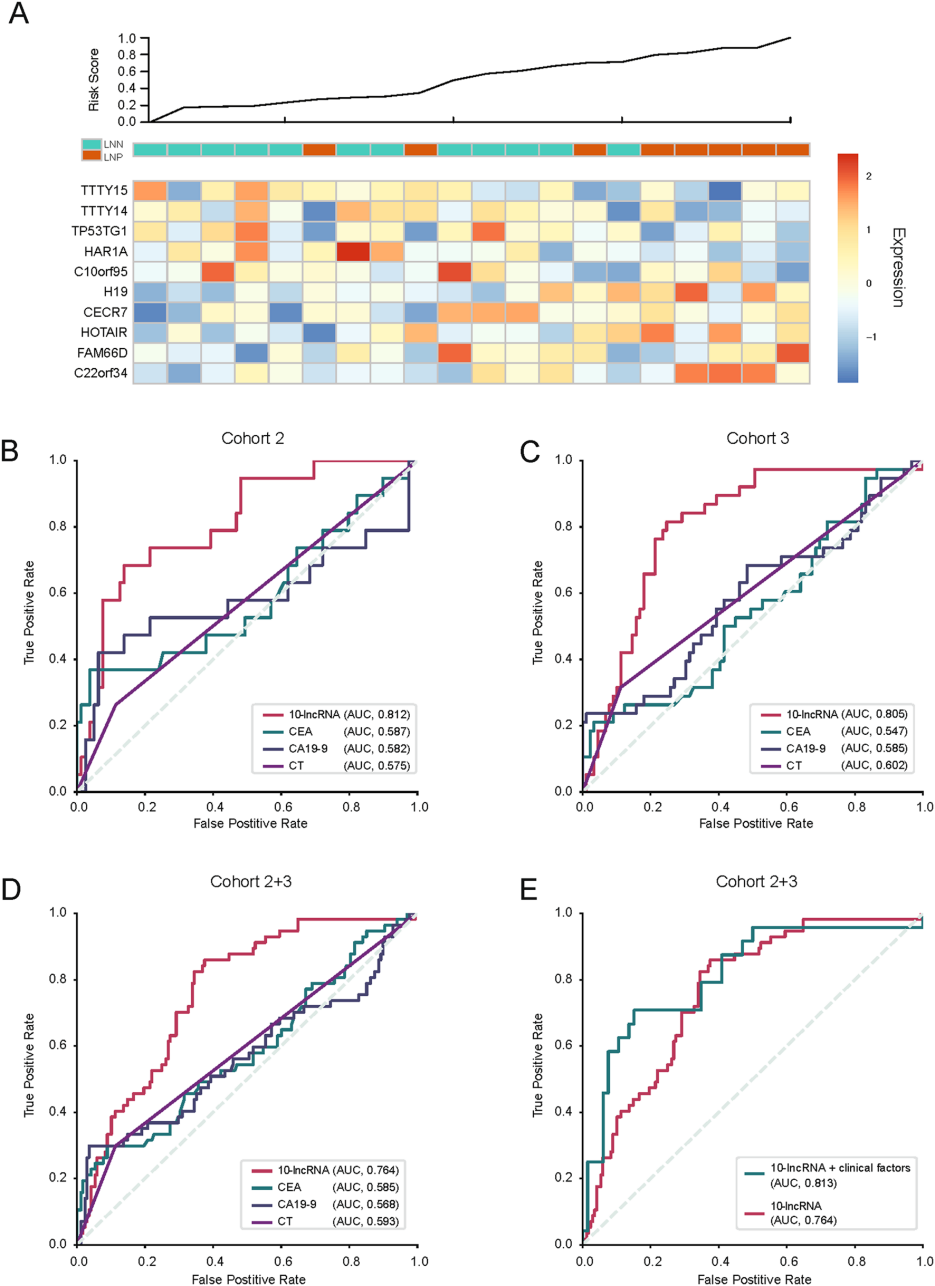
Performance of the 10-long noncoding RNA (lncRNA) panel to identify lymph node metastasis status in the clinical validation cohorts

The accuracy of diagnosis using the 10-lncRNA panel was assessed using RNA-seq in validation clinical cohort 1 and by qRT-PCR in validation clinical cohorts 2 and 3 (Table 1). All patients in cohort 1 were in T1 stage, which included 8 LNP and 12 LNN patients. The heatmap of the 10-lncRNA panel and the risk score curve are shown in Fig. 2A. As expected, there was a significant difference in the expression of corresponding lncRNAs between the LNN and LNP samples, revealing an effective diagnostic performance by our risk-prediction model (Fig. 2A).

The patients in cohort 2 and 3 had T1 (19 LNP and 79 LNN) stage and T2 (38 LNP and 89 LNN) stage GC, respectively. Multivariate LR analysis was used to assess the effectiveness of the 10-lncRNA panel in T1–T2 tumors. In cohort 2, the panel showed an AUC of 0.812 (Table 2 and Fig. 2B), and in cohort 3, the AUC was 0.805 (Table 2 and Fig. 2C). However, the predictive performance of our panel in cohort 2 + 3 (T1 + T2) was slightly reduced (AUC = 0.764, Fig. 2D) compared with verification using the individual cohorts, although there was still a good effect. This might be related to the high heterogeneity of GC and the difference in the overall expression of 10 lncRNAs in the T1 and T2 specimens. Overall, the validation results agreed with those obtained using the training cohort: the 10-lncRNA panel could robustly and effectively distinguish LNP from LNN in patients with T1 and T2 stage GC.

**Table 2:** Summary of the individual lncRNA performance to predict lymph node metastasis in clinical cohorts 2 and 3

lncRNA	Clinical cohort 2			Clinical cohort 3		
	AUC	Specificity	Sensitivity	AUC	Specificity	Sensitivity
TTTY15	0.659	0.947	0.557	0.586	0.658	0.584
TTTY14	0.526	0.368	0.949	0.656	0.579	0.753
TP53TG1	0.610	0.895	0.443	0.644	0.447	0.865
HAR1A	0.562	0.684	0.544	0.670	0.526	0.798
C10orf95	0.672	0.737	0.595	0.690	0.526	0.888
H19	0.676	0.772	0.526	0.676	0.629	0.684
CECR7	0.709	0.709	0.789	0.669	0.742	0.737
HOTAIR	0.710	0.975	0.421	0.689	0.921	0.500
FAM66D	0.701	0.709	0.842	0.707	0.843	0.632
C22orf34	0.702	0.570	0.947	0.695	0.730	0.711
Risk score	0.812	0.861	0.684	0.805	0.753	0.816

*AUC* area under the curve

A. The risk score curve and heatmap of the lncRNAs expressed between lymph node-positive (LNP) and lymph node-negative (LNN) patients in clinical cohort 1. B and C. Receiver operating characteristic (ROC) curves showing how the 10-lncRNA risk-prediction model performed in identifying lymph node (LN) metastasis compared with that of the carcinoembryonic antigen (CEA), carbohydrate antigen 19–9 (CA19-9), and computed tomography (CT) in clinical cohort 2 (B) (*p* < 0.05) and clinical cohort 3 (C) (*p* < 0.05), respectively. D. ROC curves illustrating the diagnostic value for identification of LN metastasis of the 10-lncRNA panel compared with that of CEA, CA19-9, and CT in clinical cohort 2 and cohort 3 (*p* < 0.05). E. ROC curves illustrating the diagnostic accuracy of the combinatorial model integrating the 10-lncRNA panel and clinicopathological features in clinical cohort 2 + 3 compared with that of the 10-lncRNA panel alone (*p* < 0.05).

### The 10-lncRNA panel showed better diagnostic power compared with conventional tumor markers and CT in LN metastasis

For the surgical management of patients with GC, generally, enhanced CT imaging is employed to determine clinical N stage before surgery. Typically, CT features such as an LN diameter ≥ 1 cm, ring or heterogeneous enhancement, are employed to diagnose LN metastasis. However, CT imaging cannot successfully diagnose most cases of LN metastasis, or there may be misdiagnosis; therefore, only the pathological examination of surgically excised tissue can confirm LM metastasis in such cases. As shown in Table 3, we demonstrated that our 10-lncRNA risk-prediction model could effectively identify LN metastasis using univariate and multivariate analyses, independent of preoperative clinical characteristics such as sex, age, conventional tumor markers, and CT.

**Table 3:** Univariate and multivariate logistic regression analyses of the statistical significance of the 10-lncRNA risk score to diagnose LN metastasis status in clinical cohorts 2 and 3

Variables	Univariate analysis			Multivariate analysis		
	Odds ratio	95% CI	*P*	Odds ratio	95% CI	*P*
Age	1.012	0.971–1.054	0.578	1.004	0.955–1.057	0.888
Sex	1.312	0.713–2.414	0.383	1.421	0.687–2.939	0.342
CEA	2.037	1.017–4.079	0.045	1.528	0.654–3.572	0.327
CA19-9	2.820	1.369–5.813	0.005	4.104	1.677–10.046	0.002
CT	3.333	1.588–6.997	0.001	4.073	1.661–9.991	0.002
Risk score	8.889	4.085–19.341	< 0.0001	11.072	4.706–26.046	< 0.0001

*LN* lymph node, *CI* confidence interval, *CEA* carcinoembryonic antigen, *CA19-9* carbohydrate antigen 19–9, *CT* computed tomography

To assess the diagnostic efficiency of the panel, its performance was compared with that of conventional tumor markers (CEA and CA19-9) and CT in clinical cohort. Our 10-lncRNA panel showed significant superiority over preoperative clinical factors, CEA, CA19-9, and CT (Fig. 2B, C and D, comparison of the AUC values were compared using the *DeLong* test). In addition, we combined the 10-lncRNA panel with clinicopathological features (CEA, CA19-9, and CT) in cohorts 2 + 3. The results were also encouraging: this combination further improved the diagnostic accuracy of our panel (AUC = 0.813) compared with the 10-lncRNA panel alone (Fig. 2E). In conclusion, we constructed and validated a 10-lncRNA panel that demonstrated robust discriminative power compared with current preoperative management approaches to identify cases of LNP gastric cancer.

## Discussion

Currently, minimally invasive or non-invasive, stomach-preserving, function-preserving, and individualized treatment has become a trend in global GC treatment. Clinically, determining LN status is crucial to indicate and evaluate the curative potential of GC endoscopic treatment and surgery, especially in patients with relatively early GC (T1–T2). Pathological diagnosis following radical gastrectomy remains the optimal way to evaluate a patient's GC's LN status, considering our lack of effective molecular markers that can robustly detect LN metastasis before therapeutic decision-making. Moreover, only patients with GC in situ (Tis stage) and T1a GC without LN metastasis can be treated successfully using endoscopic mucosal or submucosal resection. However, the actual LN metastasis rate of early GC (T1) is only around 20%. In addition, the incidence of regional LN metastasis is limited in patients with T2 GC, in which D2 gastrectomy might be an excessively invasive surgery, involving in a significant waste of medical resources [9, 10, 12]. Currently, the development of sentinel node navigation surgery (SNNS) and laparoscopic surgery in GC provides a direction for minimally invasive gastric surgery. The study group of the Japan Society of SNNS has already formulated the standard procedure for SNNS, which uses a dual tracer comprising technetium 99 m–labeled tin colloid and 1% isosulfan blue dye [25]. Although several single institutions have reported the successful use of SNNS, because GC has a somewhat complex lymphatic flow, there still are controversial aspects regarding the application of SNNS [12, 26, 27].

LncRNAs are mRNA-like transcripts of > 200 nucleotides with no capacity to encode proteins [25]. A variety of cancers show abnormal expression of lncRNAs, which have diverse functions in gene regulation, cell biological behavior, and tumor initiation and progression [28, 29]. To date, there have been a considerable number of studies on lymph node metastasis of GC; however, most of them explored the regulatory mechanism of a single lncRNA [30, 31]. Although these studies are meaningful and significant, the lack of a comprehensive and dynamic understanding of lymph node metastasis limits the clinical application value of these findings. The recent development and popularization of high-throughput sequencing technologies have increased our understanding of the molecular characteristics of GC [32, 33]. Notably, the different T stages of GC have strong histological heterogeneity, and the correlation between lncRNAs and LN metastasis in relatively early GC (T1–T2) remains unexplored.

In this article, we used RNA-sequencing to gain insights into the molecular biology of tumor heterogeneity and disease processes to identify LN metastasis. A systematic and comprehensive analysis of transcriptome-wide expression profiles of patients with T1–T2 GC, with and without LN metastasis, was used to establish an optimized 10-lncRNA panel to identify LN metastasis using logistic regression analysis. Subsequently, the panel was validated in three independent validation cohorts based on RNA-seq and qRT-PCR, achieving encouraging results. Our study is based on the concept of minimally invasive and non-invasive, devoted to the prediction of lymph node metastasis in early gastric cancer and clinical decision support. At the initial stage of the study, we also verified the predictive value of our panel in patients with T3 and T4 stage GC by TCGA and ACRG databases, but the results were not as expected (Supplementary Fig. 2), which may related to the heterogeneity of GC with different T stages.

Carcinoembryonic antigen (CEA) and carbohydrate antigen 19–9 (CA19-9) are the most commonly used clinical monitoring serum indicators of digestive system tumors. It has been widely reported that elevated serum CEA and CA19-9 levels correlated well with lymph node metastasis, lymphatic invasion, stage grouping, and depth of invasion [4, 34–36]. Specifically, the thresholds of protein biomarkers were set according to clinical instruction, with 5 ng/mL for CEA and 37 U/mL for CA19-9. Fan et al. also reported that elevated CEA and CA19-9 level was correlated with the presence of lymph node metastasis in early GC, but the diagnostic sensitivity of CEA and CA19-9 was not satisfactory [4]. Our further analysis demonstrated the superiority of the 10-lncRNA panel over current clinicopathological factors, including CEA, CA19-9, and CT-based imaging, to diagnose LN metastasis in patients with GC. Although the accuracy of 10-lncRNA panel in combined cohort 2 + 3 was slightly decreased, its diagnostic accuracy improved again after combining it with clinicopathological features. We also performed functional and expression enrichment analysis of the 10 lncRNAs, several of which are related to metastasis and prognosis. LncRNA *H19* is considered a carcinogenic factor in GC, and its upregulation is related to tumor cell proliferation, invasion, migration, and EMT [37]. *HOTAIR* has been reported to be related to the expression of *HER2* (encoding human epidermal growth factor receptor 2) and facilitates GC lymph node metastasis [38]. A study using TCGA-based bioinformatics analysis and microarray analysis revealed that *HAR1A* is a tumor suppressor involved in tumor progression via EMT regulation and is negatively associated with prognosis [39, 40]. In our panel, *HAR1A* also acted as a negative factor for early lymph node metastasis in GC. Similarly, *TP53TG1* and *TTTY15* have been confirmed to be differentially expressed in GC tissues compared with that in normal gastric mucosa [28, 41]. Finally, as biomarkers, each lncRNA in our panel was endowed with an additional diagnostic coefficient and made a significant contribution to the identification of LN metastasis.

This study has certain limitations. First, this was a retrospective study, and its design means that although we validated our findings in multiple clinical cohorts, prospective studies are still required. Second, the main aim of this study was to find early-stage GC biomarkers; therefore, the samples were concentrated in the T1 and T2 GC stages, which limited the sample size to discover biomarkers and had a certain impact on obtaining the panel with maximum efficiency. To overcome these limitations, larger cohorts comprising patients with GC and T1 and T2 LN metastasis are required, which might involve the participation of multiple medical institutions.

## Conclusion

Our panel provided and validated a class of biomarkers that could robustly categorize patients with relatively early GC according to their LN status prior to therapeutic decision-making, thus permitting individualized treatment. Our panel offers promising diagnostic potential to identify patients with GC with or without LN metastasis; however, our findings should be validated prospectively using clinical cohorts.

### Supplementary Information

Below is the link to the electronic supplementary material.

Supplementary file1 (DOCX 353 KB)

## Data Availability

The datasets used and analyzed during the current study are available from the corresponding author on reasonable request.

## Abbreviations

LN: Lymph node
GC: Gastric cancer
lncRNA: Long noncoding RNA
TCGA: The cancer genome atlas
ACRG: Asian cancer research group
CT: Computed tomography
AUC: Area under the curve
CEA: Carcinoembryonic antigen
CA19-9: Carbohydrate antigen 19-9
PET-CT: Positron emission tomography with CT
ESD: Endoscopic submucosal dissection
EMR: Endoscopic mucosal resection
LSNNS: Laparoscopic sentinel node navigation surgery
OS: Overall survival
DFS: Disease free survival
RNA-Seq: RNA-sequencing
LNN: Lymph node metastasis negative
LNP: Lymph node metastasis positive
TPM: Transcripts per million
LR: Logistic regression
ROC: Receiver operator characteristic
qRT-PCR: Quantitative real-time reverse transcription PCR
EMT: Epithelial–mesenchyme transition
SNNS: Sentinel node navigation surgery
