# Supplementary material for: LncRNA expression signature identified using genome-wide transcriptomic profiling to predict lymph node metastasis in patients with stage T1 and T2 gastric cancer
Source: Gastric Cancer. 2023 Sep 10;26(6):947–57. doi: 10.1007/s10120-023-01428-8 (PMC10640531; doi:10.1007/s10120-023-01428-8)
Supplement: Supplementary file 1 — Supplementary file1 (DOCX 353 KB) [file 10120_2023_1428_MOESM1_ESM.docx]

**Supplementary Information**

**Supplementary table 1. Primers used for real-time quantitative PCR analysis**

| Primers used for real-time quantitative PCR analysis | | |
| --- | --- | --- |
| LncRNA *H19* | | Forward: GCACCTTGGACATCTGGAGT |
|  |  | Reverse: TTCTTTCCAGCCCTAGCTCA |
| LncRNA *TTTY15* | | Forward:TCTATGACCTGGAAGC |
|  |  | Reverse: ATCTGATG GAACCCTA |
| LncRNA *TTTY14* | | Forward: GCCAATGGCTGTGACGGATAAGG |
|  |  | Reverse: ACCTGTTGCGGAGGACTACTGAG |
| LncRNA *TP53TG1* | | Forward: ACGAAGGTACCCAACCCTCT |
|  |  | Reverse: GGTGTAAGTGTTCGCCTGGT |
| LncRNA *HAR1A* | | Forward:TCAGCTGAAATGATGGGCGT |
|  |  | Reverse:CATCGCGGAAAACGGGATTC |
| LncRNA *HOTAIR* | | Forward: GGTAGAAAAAGCAACCACGAAGC |
|  |  | Reverse: ACATAAACCT CTGTCTGTG AGTGCC |
| LncRNA *CECR7* | | Forward:TATCAATGCCTCTGGGTGG |
|  |  | Reverse: TCCTTAGTCAGCTGGTCCT |
| LncRNA *FAM66D* | | Forward:AACCTAATCAGTTACATGAGGC |
|  |  | Reverse: GTGTTGACTTTGTTCATCTGTG |
| LncRNA *C22orf34* | | Forward:CTCCCGATCGTTCTGAGAG |
|  |  | Reverse: ATCTCTGACAGCGTCAGAG |
| LncRNA *C10orf95* | | Forward:CTGAAGGAGACAAGTCTGGA |
|  |  | Reverse: CTGTACACATACATGGTCGG |


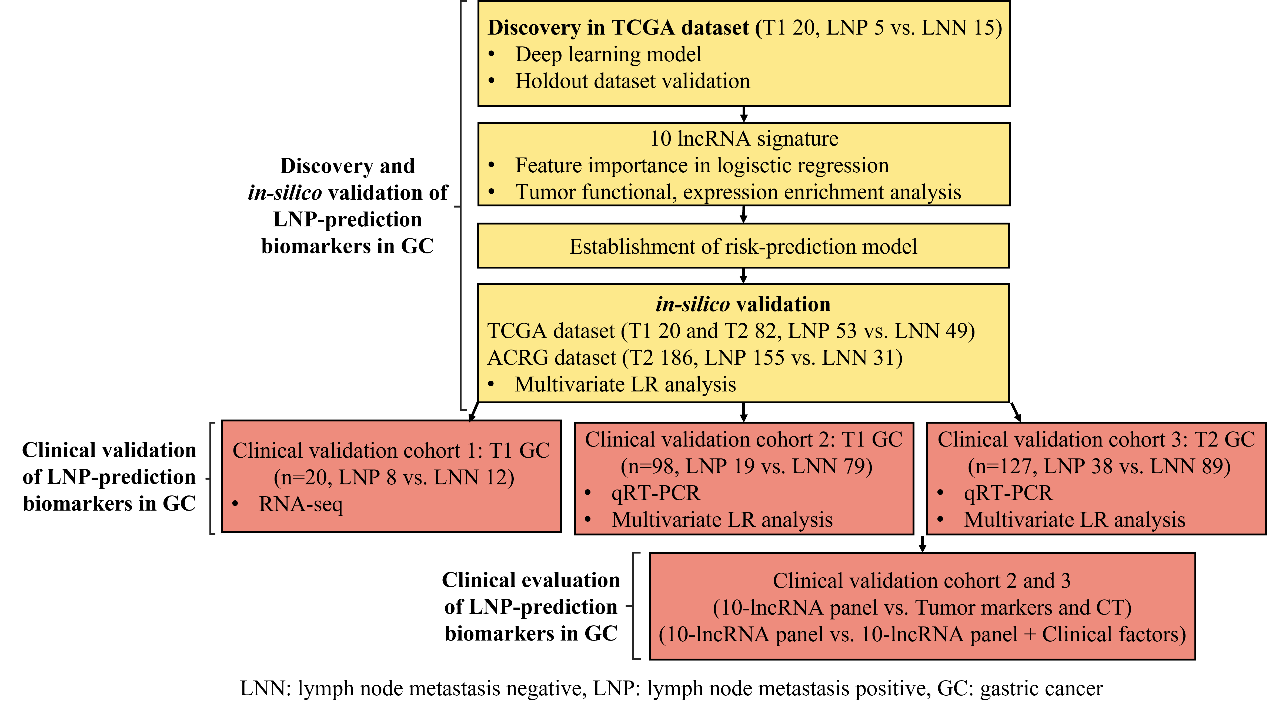


**Supplementary Figure 1: Study design and overall workflow.**

**
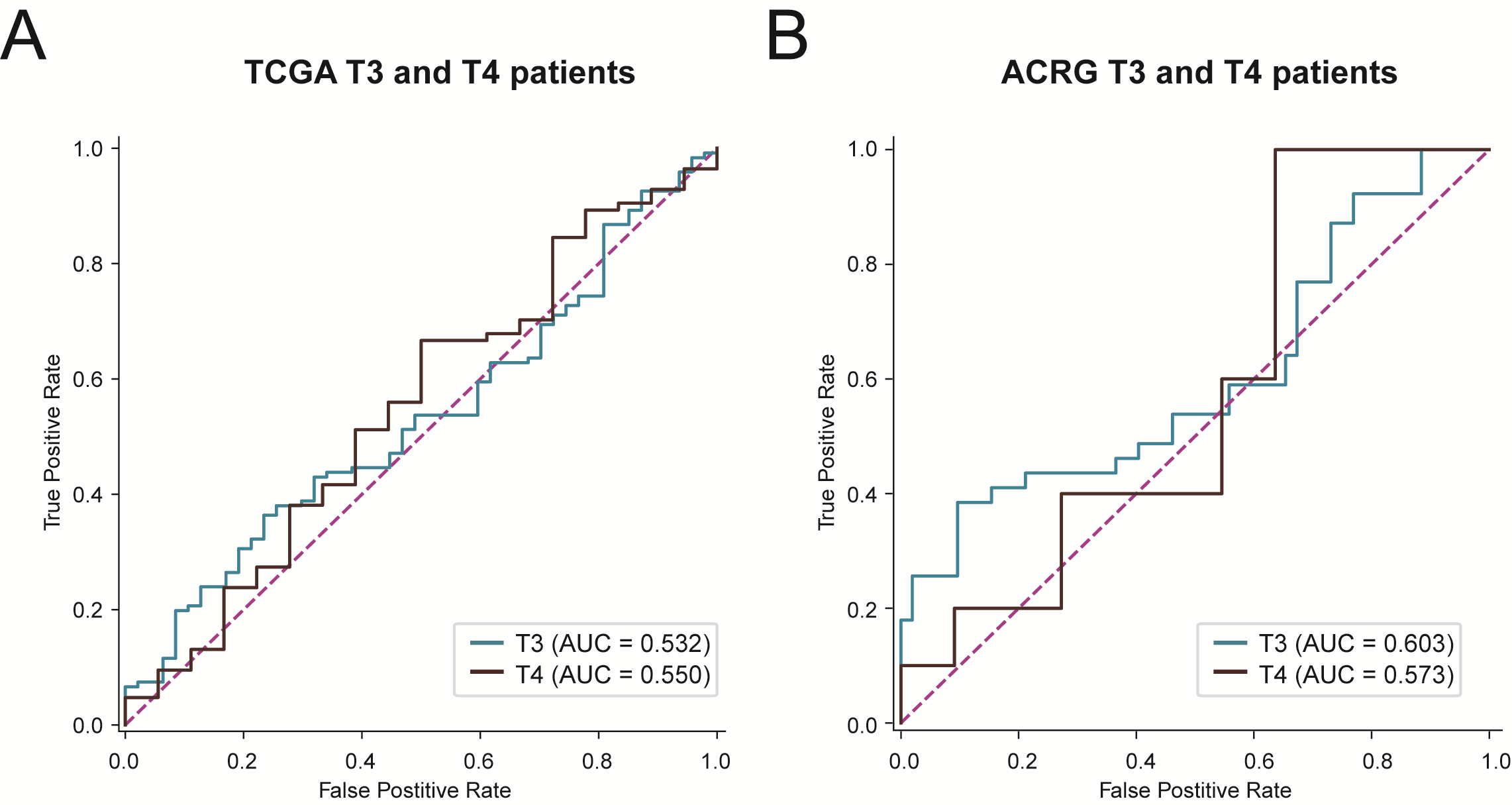
**

**Supplementary Figure 2: The long noncoding RNA expression-based signature to identify lymph node metastasis in T3 and T4 stage gastric cancer.**

A and B. Receiver operating characteristic (ROC) curves illustrating the diagnostic value for identification of lymph node metastasis of the10-lncRNA panel in T3 and T4 samples (TCGA and ACRG).
